# Neurochemical evidence supporting dopamine D1–D2 receptor heteromers in the striatum of the long-tailed macaque: changes following dopaminergic manipulation

**DOI:** 10.1007/s00429-016-1306-x

**Published:** 2016-09-09

**Authors:** Alberto J. Rico, Iria G. Dopeso-Reyes, Eva Martínez-Pinilla, Diego Sucunza, Diego Pignataro, Elvira Roda, David Marín-Ramos, José L. Labandeira-García, Susan R. George, Rafael Franco, José L. Lanciego

**Affiliations:** 1 Department of Neurosciences, Center for Applied Medical Research (CIMA), University of Navarra, Pio XII Avenue 55, 31008 Pamplona, Spain; 2 Centro de Investigación Biomédica en Red sobre Enfermedades Neurodegenerativas (CIBERNED), Madrid, Spain; 3 Department of Morphological Sciences, University of Santiago de Compostela, Santiago De Compostela, Spain; 4 Campbell Family Mental Health Research Institute, Centre for Addiction and Mental Health, Departments of Medicine and Pharmacology, University of Toronto, Toronto, ON Canada; 5 Department of Biochemistry and Molecular Biology, University of Barcelona, Barcelona, Spain

**Keywords:** Basal ganglia, Putamen, Caudate nucleus, Dyskinesia, MPTP

## Abstract

Although it has long been widely accepted that dopamine receptor types D1 and D2 form GPCR heteromers in the striatum, the presence of D1–D2 receptor heteromers has been recently challenged. In an attempt to properly characterize D1–D2 receptor heteromers, here we have used the in situ proximity ligation assay (PLA) in striatal sections comprising the caudate nucleus, the putamen and the core and shell territories of the nucleus accumbens. Experiments were carried out in control macaques as well as in MPTP-treated animals (with and without dyskinesia). Obtained data support the presence of D1–D2 receptor heteromers within all the striatal subdivisions, with the highest abundance in the accumbens shell. Dopamine depletion by MPTP resulted in an increase of D1–D2 density in caudate and putamen which was normalized by levodopa treatment. Two different sizes of heteromers were consistently found, thus suggesting that besides individual heteromers, D1–D2 receptor heteromers are sometimes organized in macromolecular complexes made of a number of D1–D2 heteromers. Furthermore, the PLA technique was combined with different neuronal markers to properly characterize the identities of striatal neurons expressing D1–D2 heteromers. We have found that striatal projection neurons giving rise to either the direct or the indirect basal ganglia pathways expressed D1–D2 heteromers. Interestingly, macromolecular complexes of D1–D2 heteromers were only found within cholinergic interneurons. In summary, here we provide overwhelming proof that D1 and D2 receptors form heteromeric complexes in the macaque striatum, thus representing a very appealing target for a number of brain diseases involving dopamine dysfunction.

## Introduction

Up to five distinct types of dopamine receptors (from D1 to D5) have been currently described and often grouped in two categories, one made of D1 and D5 receptors, the other one comprising D2, D3 and D4 receptors. These dopamine receptors are G protein-coupled receptors (GPCRs) that mediate cyclic-AMP-dependent signaling (Beaulieu et al. 2015). In the past few years, increasing evidence suggested that dopamine receptors—as well as many other classes of GPCRs—are likely to form heteromeric complexes that represent novel molecular entities with properties distinct from those of each component receptor, when considered separately. Indeed, it might be argued that dopamine receptors have a natural tendency to form heteromeric complexes, both between themselves as well as with GPCR receptors other than dopaminergic. Considering GPCR heteromers made of D1 and D2 receptors, the generation of a new phospholipase C-mediated calcium signal following D1 and D2 coactivation was reported by Lee et al. (2004). Later on, cumulative evidence showed that D1–D2 receptor heteromers represent a novel molecular entity with unique molecular, pharmacological and electrostatic interactions with activation of CaMKII and BDNF in rat striatum (So et al. 2005; Dziedzicka-Wasylewska et al. 2006; Rashid et al. 2007; Hasbi et al. 2009; Ng et al. 2010; Verma et al. 2010; Perreault et al. 2011, 2012; Pei et al. 2010; O’Dowd et al. 2012). Furthermore, and besides D1–D2 receptor heteromers, a number of dopaminergic heteromeric complexes have been described, comprising D1/D3 (Marcellino et al. 2008; Fiorentini et al. 2008, 2010; Farré et al. 2015), D2/D3 (Scarselli et al. 2001), D2/D4 (Borroto-Escuela et al. 2011; González et al. 2012) and D2/D5 (So et al. 2009). Considering non-dopaminergic partners, GPCR heteromers made of dopamine D1 and adenosine A1 receptors have been described (Ginés et al. 2000; Shen et al. 2013), and the same holds true for D1 and histamine H3 receptors (Ferrada et al. 2009; Moreno et al. 2011a), D1 and mu opioid receptor (Juhasz et al. 2008), D1 and galanin 1 receptor (Moreno et al. 2011b), D1 and CRH-2A (Fuenzalida et al. 2014) and for D1 and ghrelin GHS-R1a receptor (Jiang et al. 2006; Schellenkens et al. 2013). Regarding D2 receptors, heteromers were described together with adenosine 2A receptors (Hilion et al. 2002; Navarro et al. 2008; Borroto-Escuela et al. 2010; Pinna et al. 2014; Bonaventura et al. 2014), cannabinoid CB1 receptors (Navarro et al. 2008; Pinna et al. 2014; Bonaventura et al. 2014; Przybyla and Watts 2009), Histamine H3 (Ferrada et al. 2008), and with serotonin 2A receptors (Borroto-Escuela et al. 2010; Lukasiewicz et al. 2010, 2011).

For most of the GPCR heteromers described above, the presence of molecular interactions between each component receptor was properly addressed ex vivo and in vitro using a number of complementary techniques, these including co-immunoprecipitation, BRET, FRET and competition binding ligation assays. However, it is also worth noting that these procedures can, overall, be viewed as indirect indications on the presence of GPCR heteromers. In this regard, the recent introduction of a new technique known as in situ proximity ligation assay (PLA; Söderberg et al. 2008) has represented a major step forward for the accurate visualization of GPCR heteromers in native tissues. Initially designed to detect protein-to-protein interactions, the PLA technique enabled for the first time the accurate visualization of GPCR heteromers both at the in vitro and ex vivo levels. Indeed, the precise location of a number of GPCR heteromers has been made available by taking advantage of the PLA technique, these including D2/D4 receptor heteromers (Borroto-Escuela et al. 2011), D2/A2A (Navarro et al. 2008; Bonaventura et al. 2014; Trifilieff et al. 2011), D1/D3 (Farré et al. 2015), D2 and angiotensin type II receptors (Martinez-Pinilla et al. 2015), cannabinoid CB1 and CB2 receptors (Sierra et al. 2015), as well as CB1 and GPR55 receptors (Martinez-Pinilla et al. 2014).

The well-known presence of D1–D2 receptor heteromers in striatal neurons has been recently challenged (Frederick et al. 2015). Using a number of in vitro and ex vivo techniques, together with behavioral analysis, Frederick et al. (2015) have failed to find any conclusive evidence supporting the presence of these heteromers. Most importantly, the PLA technique was used to provide the final proof of the lack of D1–D2 heteromers. Here, we have used what we think is the most appropriate variant procedure of the PLA technique to adequately assess the presence of D1–D2 receptor heteromers in the striatum of *Macaca fascicularis*. Experiments were carried out in control as well as in MPTP-treated macaques (with and without levodopa-induced dyskinesia). Contrary to what was suggested by Frederick et al. (2015), our results clearly indicate the presence of D1–D2 receptor heteromers in all striatal territories of the macaque, these including the caudate nucleus, the putamen and the core and shell of the nucleus accumbens. Furthermore, a number of changes in the densities for D1–D2 heteromers within striatal territories as well as in different clinical states were also found, together with the first ultrastructural evidence accounting for the presence of D1–D2 heteromers. Finally, striatal projection neurons and interneurons showing different types of D1–D2 heteromers were also properly identified.

## Materials and methods

A total of six naïve young adult male *Macaca fascicularis* primates (body weight 3.4–4.3 kg) were used in this study. Animal handling was conducted in accordance with the European Council Directive 2010/63/UE as well as in keeping with Spanish legislation (RD53/2013). The experimental design was approved by the Ethical Committee for Animal Testing of the University of Navarra (ref: 009-12). All animals were captive-bred and supplied by R.C. Hartelust (Leiden, The Netherlands).

**Fig. 1:**
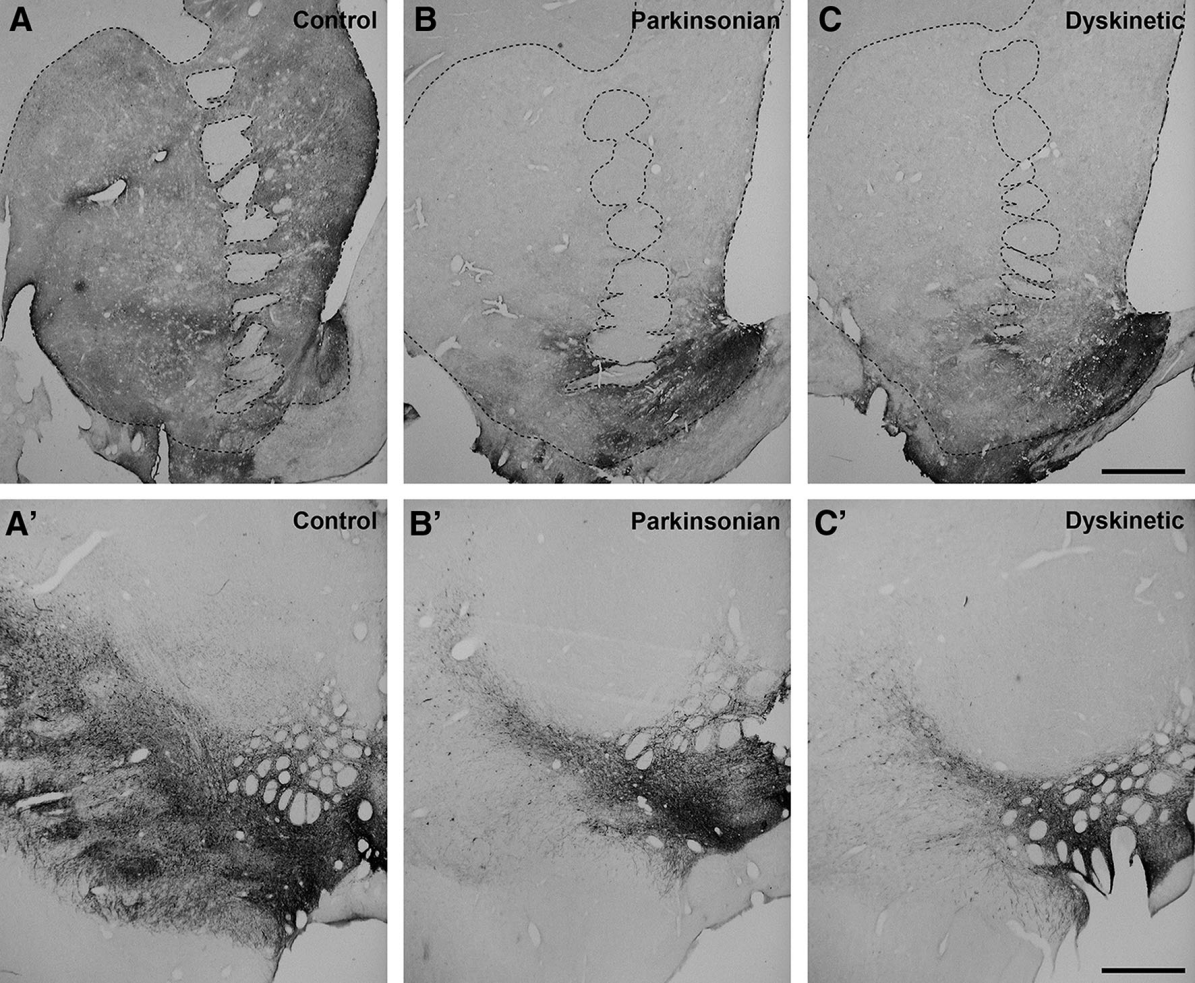
Pattern of nigrostriatal denervation following MPTP treatment. Coronal sections taken through the striatum (**a**–**c**) and the substantia nigra (**a**′–**c**′) stained for tyrosine hydroxylase (TH). In keeping with existing knowledge, neurons located in the ventral tegmental area (VTA) are by far less sensitive to MPTP when compared to dopaminergic neurons from the substantia nigra pars compacta (SNc). In other words, the MPTP treatment resulted in an almost complete depletion of dopaminergic terminals in both the caudate and putamen nuclei, whereas the dopaminergic innervation of the core and shell territories of the nucleus accumbens (VTA-recipient areas) is better preserved. *Scale bar* 2 mm for *panels* **a**–**c** and 1 mm for *panels* **a**′–**c**′

The dopaminergic neurotoxicant 1-methyl-4-phenyl-1,2,3,6-tetrahydropyridine (MPTP; Sigma) was administered intravenously to four macaques at a concentration of 0.2 mg/kg (injected once weekly) until animals reached a stable Parkinsonian syndrome. The severity of the MPTP-induced Parkinsonism was evaluated weekly by two independent blind observers using a modified UPDRS clinical rating scale (Kurlan et al. 1991) where the highest score was 29. All MPTP-treated macaques reached a stable score between 21 and 23 points that was maintained over a period of 2 months of MPTP washout. Two monkeys were selected to receive daily oral treatment with levodopa and benserazide (25 mg/kg of Madopar, Roche, France). These monkeys developed overt dyskinetic symptoms after the first month of treatment and remained stable until the stereotaxic injection of biotinylated dextran amine (BDA; see below). The extent of the MPTP-induced dopaminergic depletion was assessed by the immunohistochemical detection of tyrosine hydroxylase (Fig. 1).

### Tracer delivery, perfusion and histological processing

Surgical anesthesia was induced by intramuscular injection of ketamine (5 mg/kg) and midazolam (5 mg/kg). Local anesthesia was implemented just before surgery by means of a 10 % solution of lidocaine. Analgesia was achieved with a single intramuscular injection of flunixin meglumine (Finadyne, 5 mg/kg) delivered at the end of the surgical procedure and repeated 24 and 48 h post-surgery. A similar schedule was conducted for antibiotic delivery (ampicillin, 0.5 ml/day). After surgery, animals were kept under constant monitoring in single cages with ad libitum access to food and water.

**Fig. 2:**
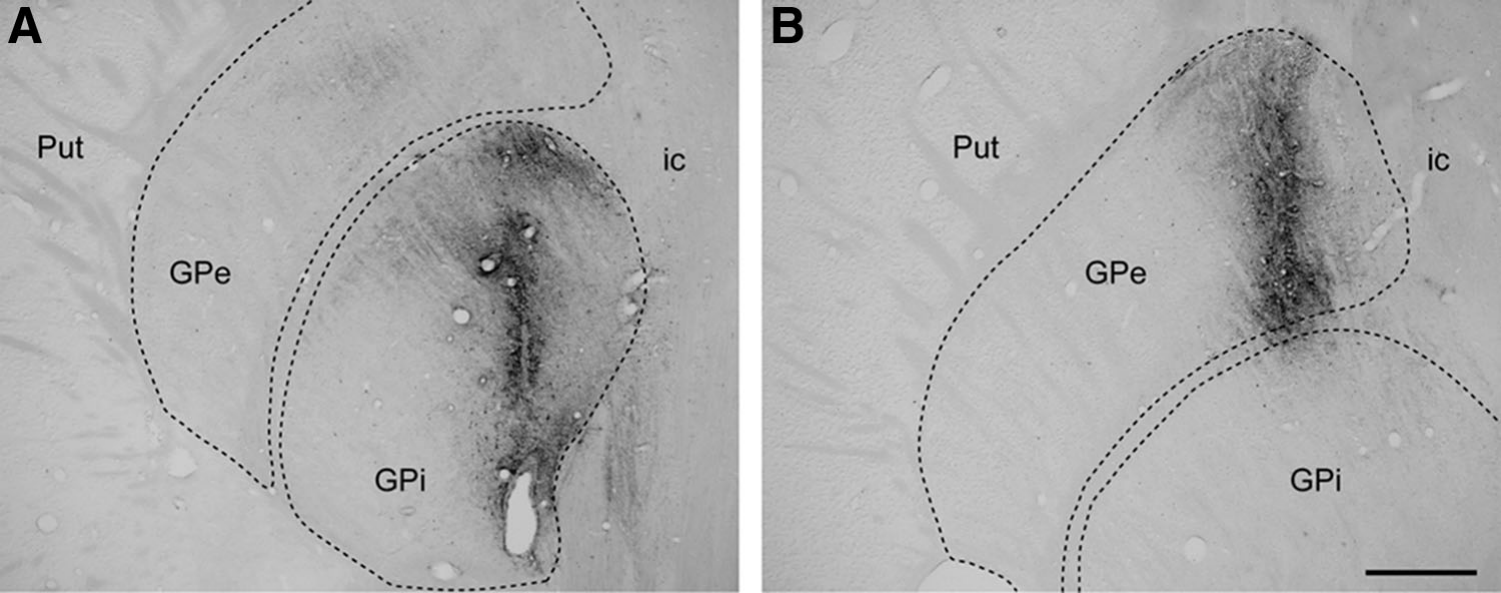
BDA injection sites. Representative examples of the injection sites for BDA in the GPi (**a**) and in the GPe (**b**). It is worth noting that both injections remained within the boundaries of the targeted nuclei, without any tracer leakage through the injection tract. *Scale bar* 1 mm in both *panels*

Stereotaxic coordinates for the internal and external divisions of the globus pallidus (GPi and GPe, respectively) were taken from a *M. fascicularis* stereotaxic atlas (Lanciego and Vázquez 2012). During surgery, target selection was assisted by ventriculography. Coordinates for the GPi nucleus were 3.5 mm caudal to the anterior commissure (ac), 1.5 mm ventral to the bicommissural plane (ac–pc plane) and 6 mm lateral to the midline. Coordinates for the GPe nucleus were 3.5 mm caudal to the ac, 1.5 mm dorsal to the ac–pc plane and 8.5 mm lateral to the midline. Representative examples of the injection sites are shown in Fig. 2.

All monkeys (two control, two Parkinsonian and two dyskinetic animals) received a single pressure injection of 1 ml of biotinylated dextran amine (BDA; 10 kDa, lysine fixable, ref: D-1956 from Molecular Probes-Invitrogen) through a Hamilton syringe (5 mg/mL in 0.01 M phosphate buffer, pH 7.5) in the GPi or GPe nuclei. Tracer delivery was accomplished in pulses of 0.1 ml/2 min, and once completed, the microsyringe was left in place for 15 min before withdrawal to minimize tracer reflux through the injection tract.

Two weeks post-surgery, animals were anesthetized with an overdose of 10 % chloral hydrate and perfused transcardially (for dyskinetic monkeys, terminal anesthesia was administered at the time point at which they showed overt, peak-of-dose dyskinesia). The perfusates consisted of a saline Ringer solution followed by 3000 ml of a fixative solution containing 4 % paraformaldehyde and 0.1 % glutaraldehyde in 0.125 M phosphate buffer (PB), pH 7.4. Perfusion was continued with 1000 ml of a cryoprotectant solution made of 10 % glycerin and 1 % dimethyl sulfoxide (DMSO) in 0.125 M PB, pH 7.4. Once perfusion was completed, the skull was opened, the brain removed and stored for 48 h in a cryoprotectant solution containing 20 % glycerin and 2 % DMSO in 0.125 M PB, pH 7.4. Finally, frozen serial coronal sections (40 mm-thick) were obtained on a sliding microtome and collected in 0.125 M PB, pH 7.4, as 10 series of adjacent sections. These series were used for (1) immunohistochemical detection of tyrosine hydroxylase, (2) histochemical detection of transported BDA, later counterstained with cresyl violet, (3) immunofluorescent detection of D1–D2 heteromers using the PLA technique counterstained with Topro-3, (4) fluorescent detection of transported BDA combined with PLA detection of D1–D2 heteromers, (5) immunofluorescent detection of cholinergic interneurons combined with PLA stain for D1–D2 and counterstained with Topro-3, (6) immunofluorescent detection of parvalbumin-positive interneurons combined with PLA stain for D1–D2 and counterstained with Topro-3, (7) immunofluorescent detection of nNOS-positive interneurons combined with PLA stain for D1–D2 and counterstained with Topro-3, (8) immunofluorescent detection of calretinin-positive interneurons combined with PLA stain for D1–D2 and counterstained with Topro-3, (9) control stains for assessing the specificity of the PLA stain for D1–D2 and (10) PLA-based ultrastructural detection of D1–D2 heteromers.

### Detection of transported BDA

Histochemical detection of transported BDA was carried out on coronal sections throughout the entire rostrocaudal extent of the left hemisphere. Sections were incubated in HRP-conjugated streptavidin (1:5000; 90 min; Sigma) and finally visualized in blue–black with a nickel-enhanced solution of DAB (Sigma) Sections were mounted on gelatin-coated slides, air-dried and finally coverslipped with Entellan (Merck).

### In situ proximity ligation assay (PLA)

Here, we have used a rat anti-D1 receptor antibody (1:200; ref: D2944; Sigma) and a rabbit anti-D2 antibody (1:100; ref: AB5084P; Millipore). Specificity of the antibodies against dopamine receptors D1 and D2 was tested previously (Lee et al. 2004) and validated in D1- and D2- gene-deleted mice (Perrreault et al. 2010). Furthermore and related to the anti-D2 antibody, the immunogen peptide has no significant homology with other subtypes of dopamine receptors, and the specificity of this antibody against the immunogen peptide was shown by Boundy et al. (1993). This anti-D2 antibody was routinely used for the characterization of D2-expressing striatal neurons (Wang and Pickel 2002; Deng et al. 2006).

Although the most standard way of using the PLA technique is to rely on the so-called “secondary detection”, i.e., using the Duolink II in situ PLA detection kit (Sigma-Olink Bioscience), that is made of secondary antibodies tagged to complementary DNA probes plus and minus, here the most appropriate choice is to create dedicated PLA probes using the Duolink in situ Probemaker kit (Sigma-Olink Bioscience). This is because plus and minus PLA probes made in mouse, rabbit and goat are the only ones available within the Duolink II in situ PLA detection kits. In other words, anti-rat PLA probes (plus or minus) are not available from Sigma-Olink Bioscience. Accordingly, to create the PLA probes used here, we have conjugated the rat anti-D1 antibody with a MINUS oligonucleotide (Duolink in situ Probemaker MINUS, ref: DUO92010, Sigma-Olink) and the rabbit anti-D2 antibody with a PLUS oligonucleotide (Duolink in situ Probemaker PLUS, ref: DUO92009, Sigma-Olink) following the guidelines supplied by the manufacturer. After incubation for 1 h at 37 °C with the blocking solution in a preheated humidity chamber, sections were incubated overnight with these PLA probe-linked primary antibodies (final concentration of 65 mg/ml) at 4 °C. After washing with buffer A (ref: DUO82047, Sigma-Olink) at room temperature (RT), sections were incubated with Topro-3 (1:400, 1 h at 37 °C; ref: T3605; Invitrogen). Next, sections were incubated with the ligation solution (ref: DUO92008, Sigma-Olink) for 1 h at 37 °C in a humidity chamber. Sections were then washed with buffer B (ref: DUO82048, Sigma-Olink), followed by a wash with buffer B x 0.01. Samples were mounted using an aqueous mounting medium. Appropriate negative control assays were carried out to ensure that there was a lack of nonspecific labeling and amplification.

Statistical analyses on the D1–D2 receptor heteromers density were conducted using dedicated software known as Duolink ImageTool (ref: DUO90806, Sigma-Olink). This software has been developed for quantification of PLA signals and cell nuclei in images generated from fluorescence microscopy. Briefly, for each striatal territory (caudate, putamen, accumbens core and shell; 5–6 sections per animal) and for each animal (control, parkinsonian and dyskinetic), a stack of two channels (one for D1–D2 PLA stain and another one for TOPRO stain) and 15 Z stacks with a step size of 0.43 mm each covering an area of 104.1 mm^2^ were randomly acquired with a 63× oil-immersion lens (N.A. 1.3). For the automatic analysis, neurons were differentiated from glial cells by the exclusion of cells with nuclear size smaller than 15 mm. Although some small neurons may have been excluded, this criterion reliably excludes all non-neuronal cells. The data in the graphs are presented as the mean ± SEM. Statistical analysis was performed with SPSS 18.0 software. A one-way ANOVA was used to evaluate the fit of the data to a normal distribution and then, Student’s t test was used to evaluate paired comparisons. Significant differences were considered when *p* < 0.05.

The ultrastructural detection of D1–D2 receptor heteromers was carried out using the PLA technique followed by immunogold labeling and silver enhancement (Sierra et al. 2015). Briefly, free-floating sections including the caudate, putamen and core and shell territories of the Acb nucleus were incubated 15 min in a 0.1 % sodium borohydride solution. After rinsing in PB and buffer A, sections were incubated for 1 h at 37 °C with the blocking solution followed by overnight incubation at 4 °C with the PLA probe-linked antibodies described above. D1–D2 receptor heteromers were detected using the Duolink II in situ PLA brightfield detection kit (ref: DUO92012, Sigma-Olink). Next, sections were washed with buffer A at RT and incubated with the ligation solution for 1 h at 37 °C. After washing with buffer A, sections were incubated with the amplification solution for 100 min at 37 °C. Next, sections were incubated with the detection solution, made of peroxidase-labeled oligonucleotides for 1 h at RT. After several rinses in buffer A, free-floating sections were incubated in a blocking solution containing 3 % NGS, 0.005 % Triton X-100, 1 % BSA, 0.05 M glycine and 1 % w/v nonfat dry milk in PBS for 1 h. Next, sections were incubated overnight at 4 °C with goat anti-peroxidase 4 nm colloidal gold (ref: 123-185-021, Jackson Immunoresearch) diluted 1:100 in a solution of 3 % NGS, 0.005 % Triton X-100, 1 % BSA and 1 % w/v nonfat dry milk in PBS. Sections were then washed in 0.1 M PB and postfixed in 2.5 % glutaraldehyde for 2 h. After a number of rinsing steps with 0.1 M PB and distilled water, sections were incubated for 90 min at RT in a silver enhancement solution (ref: 500.044, Aurion E-Gent SE-EM Silver Enhancement Reagents) and finally postfixed in a 1 % solution of osmium in distilled water for 20 min. Next, sections were dehydrated in 50 % ethanol (2 × 10 min), 1 % uranyl acetate in 20 % ethanol (45 min), followed by 90 % ethanol, 100 % ethanol and propylene oxide (2 × 10 min each) and incubated sequentially with 3:1, 1:1, 1:3 propylene oxide and Embed-812 mix (30 min each) followed by overnight incubation at RT in straight Embed-812. Sections including representative territories of the post-commissural putamen and caudate nucleus were flat-embedded and baked in 60 °C oven for 72 h. Following polymerization, regions of interest were checked with low-magnification lenses and removed using a scalpel. Selected fragments were glued onto polymerized resin specimen blocks and stored at 4 °C. Using a Leica Ultracut R ultramicrotome, thin sections of silver–gold color were collected on carbon-coated grids (150 mesh) and stored until use. Grids were examined using a digital Zeiss Libra energy filter transmission microscope (EFTEM) operated at 80,000 kV.

**Fig. 3:**
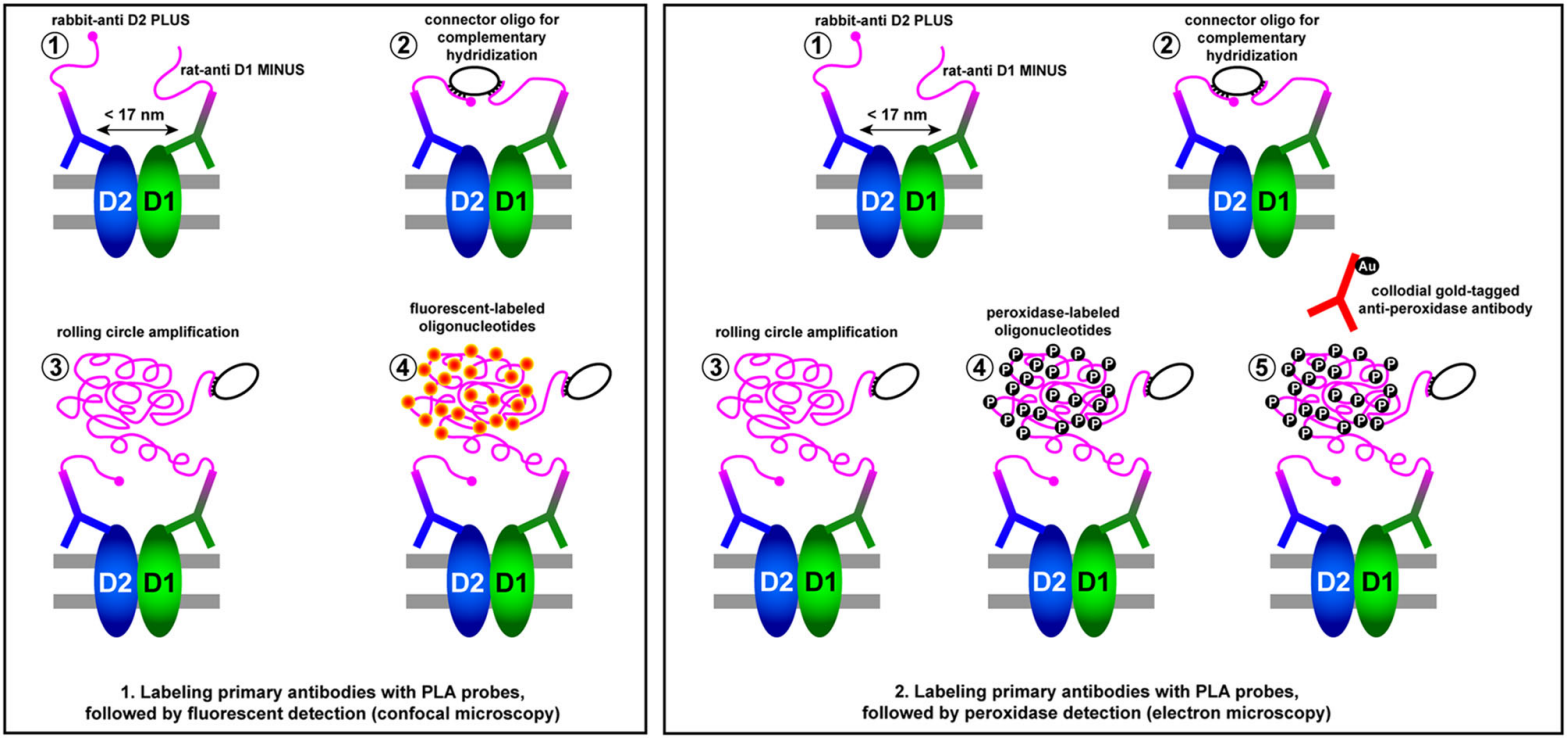
Schematic representation of the conducted protocol for the in situ proximity ligation assay (PLA). Two Duolink Probemaker kits were used to provide both species-specific primary antisera (rabbit anti-D2 and rat anti-D1) with complementary DNA strands (plus and minus probes). When the primary antisera are in close proximity, the DNA strands can interact through the addition of connector oligonucleotides, later amplified via rolling circle amplification. Next, either fluorescent dye- or peroxidase-labeled complementary oligonucleotides are added, enabling the visualization of the D1–D2 receptor heteromer by fluorescent and electron microscopy, respectively

A schematic representation of the conducted PLA procedures for confocal and electron microscope examination is provided in Fig. 3.

### Combinations of the PLA stain with neuronal markers

To properly assess the identities of striatal neurons expressing D1–D2 receptor heteromers, a number of markers were used to ascertain different populations of striatal interneurons and BDA-labeled projection neurons. In all cases, the PLA technique was carried out first, followed by either the immunofluorescent stain of striatal interneurons or the histochemical detection of BDA-labeled neurons. Cholinergic neurons were detected using a goat anti-ChAT primary antibody (1:1000; ref: AB144P, Millipore) followed by an Alexa488-coupled donkey anti-goat IgG (1:200; ref: A-11055, Thermo Scientific). A rabbit anti-parvalbumin antibody (1:250; ref: AB15736, Millipore) was used to disclose parvalbumin-positive neurons, followed by an Alexa488-coupled donkey anti-rabbit IgG (1:200, ref: A-21206, Thermo Scientific). Calretinin-containing neurons were detected with a goat anti-calretinin antibody (1:500; ref: AB1550, Millipore) followed by an Alexa488-coupled donkey anti-goat IgG (1:200). Finally, for nNOS-positive neurons, a rabbit anti-nNOS antibody (1:500; ref: AB5380, Millipore) was used followed by an Alexa488-coupled donkey anti-rabbit IgG (1:200). Striatofugal neurons retrogradely labeled with BDA were visualized following incubation with an Alexa488-coupled streptavidin (1:100; ref: S32354, Molecular Probes-Invitrogen).

## Results

### Striatal expression of D1–D2 receptor heteromers

**Fig. 4:**
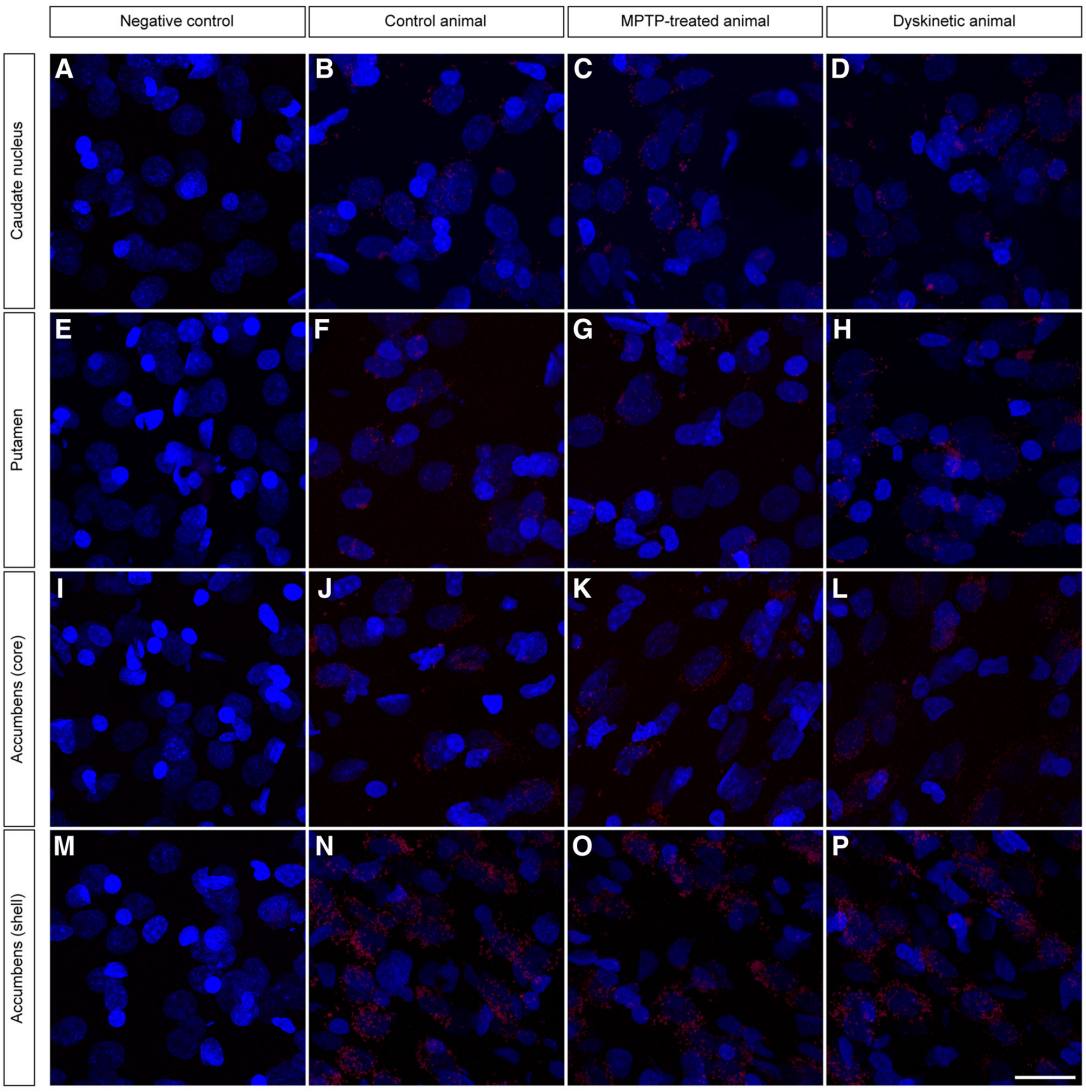
PLA-based detection of D1–D2 receptor heteromers. The use of the PLA protocol enabled the unequivocal detection of D1–D2 receptor heteromers in all striatal territories (caudate nucleus, putamen and the core and shell subdivisions of the accumbens) across all animal groups (control, MPTP-treated and dyskinetic animals). Negative control experiments, conducted the same way by avoiding the conjugation of the anti-D2 antibody with the Duolink Probemaker kit, resulted in a complete lack of stain. Each D1–D2 receptor heteromer is visualized as a single red spot. Cell nuclei are counterstained with Topro-3 (*blue color*). The strongest PLA stain is typically observed in the shell of the nucleus accumbens, followed by a gradual decline when considering the core of the accumbens, the putamen and the caudate nucleus. This pattern of staining is maintained in all the different animal groups. *Scale bar* 20 mm in all *panels*

The PLA assay revealed the presence of D1–D2 receptor heteromers in neurons located in the caudate nucleus (CN), the putamen (Put) and the core and shell subdivisions of the nucleus accumbens (AcbCo and AcbSh, respectively) in all animal groups: (1) control, (2) MPTP-treated (parkinsonian) and (3) MPTP- and levodopa-treated (dyskinetic animals). The specificity of the conducted PLA stain is exemplified by the fact that D1–D2 receptor heteromer expression was observed as red spots surrounding Topro-counterstained nuclei of striatal neurons, without any PLA signal when considering glial cells. It is also worth noting that negative control experiments (performed the same way as the conducted PLA assays but removing the conjugation of the anti-D2 primary antibody with the Duolink in situ Probemaker PLUS kit) showed a complete lack of stain, both in neurons and glial cells (Fig. 4).

**Fig. 5:**
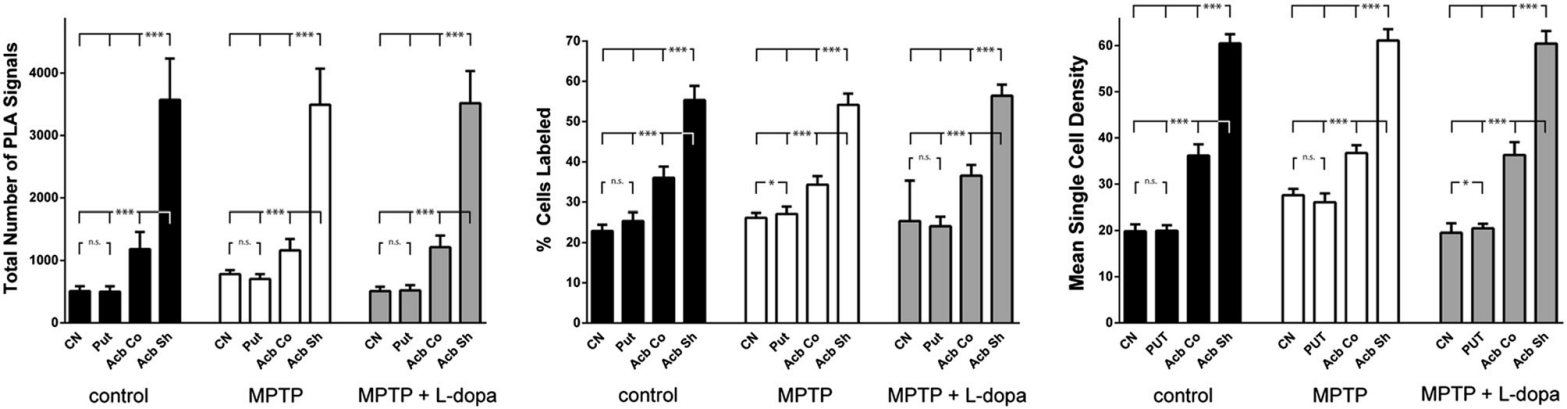
Histograms showing the obtained quantification of PLA signals. Up to three parameters were considered, comprising (i) the total number of D1–D2 receptor heteromers (reflecting total number of PLA signals), (ii) the percentage of cells showing PLA labeling vs. those lacking PLA stain and (iii) the mean single-cell densities for observed PLA signals. When comparing intra-group data, both the shell and core subdivisions of the accumbens showed highly significant changes for all the parameters (*p* < 0.001). Moreover, the total number of PLA signals failed to reach statistical significance between the caudate and putamen nucleus in the three animal groups. This also applies for comparisons between the caudate and putamen in control and dyskinetic animals (percentage of labeled cells) as well as when comparing mean single-cell densities in control and MPTP-treated animals. When considering the percentage of labeled cells in the MPTP-treated group, and besides comparisons made between the core and shell subdivisions, small significant changes were observed between the caudate and putamen (*p* = 0.018). This also holds true when comparing the mean single-cell densities at the level of the caudate and putamen in the dyskinetic group (*p* = 0.023). Although the relatively small number of animals per group (*n* = 2) impeded inter-group comparisons, there is a tendency showing a mild increase in the total number of D1–D2 heteromers in the MPTP-treated group, reflecting a moderate increase in the mean single-cell densities without any change in the percentage of labeled cells. It is also worth noting that in dyskinetic animals, all values returned back to control conditions as a result of the chronic treatment with levodopa

When considering the total number of D1–D2 receptor heteromers, the conducted statistical analysis revealed the highest expression in AcbSh neurons, followed by a gradual decline in intensity within the AcbCo, the Put and the CN. This expression pattern was maintained across all animal groups (Fig. 5). Although statistically significant differences were observed between several different territories within each individual animal group (i.e., intra-group differences), the relatively small number of animals in our sample (two animals per group) impeded inter-group statistical comparisons. Nevertheless, there is a clear tendency for increased D1–D2 expression levels in the caudate nucleus and putamen when considering the group of MPTP-treated primates, whereas expression levels between control cases and dyskinetic animals were found to be roughly similar. Furthermore, important differences were found across regions when analyzing the percentage of neurons showing or not showing PLA labeling. In this regard, and considering the CN and Put nuclei (those with lower expression of D1–D2 receptor heteromers) in control animals, on average, up to 23 % of neurons in the CN showed expression of D1–D2 receptor heteromers, together with 25 % of labeled neurons for the Put nucleus. At the level of the AcbCo, the percentage of neurons with D1–D2 receptor heteromers was estimated to be 36 %, whereas 55 % of neurons in the AcbSh showed PLA stain (Fig. 5). Moreover, significant statistical differences were found when analyzing the mean intensity of D1–D2 receptor heteromer expression at the single-cell level across different striatal territories. In this regard, the mean single-cell density for D1–D2 receptor heteromers was estimated to be of 19.8 in the CN, 19.9 in the Put nucleus, 36.1 in the AcbCo and 60.4 in the AcbSh (Fig. 5). In summary, the CN is characterized for the lowest number of D1–D2 receptor heteromers, together with the smallest percentage of labeled neurons, these cells also showing the lowest D1–D2 receptor heteromer density. By contrast, the AcbSh accounted for the highest abundance of D1–D2 receptor heteromers, not only in terms of the total number of receptor heteromers, but also showing the highest percentage of labeled neurons and the highest D1–D2 densities within labeled cells. When considering the MPTP-treated animal group (without dyskinesia), e.g., the one showing the highest expression levels for D1–D2 receptor heteromers, the obtained percentages of labeled neurons were 26 % for CN, 27 % for Put, 34 % for AcbCo and 54 % for AcbSh. At the single-cell level, obtained densities for D1–D2 receptor heteromers were 27.6 for CN, 26.1 for Put, 36.7 for AcbCo and 61.1 for AcbSh. Although the percentages of labeled neurons are similar to control animals for all striatal territories, there is a moderate increase in receptor heteromer densities for neurons located in CN and Put, whereas the AcbCo and AcbSh territories showed similar values for both percentages of labeled neurons and mean single-cell densities. Obtained values for dyskinetic animals were roughly similar to those of control monkeys in terms of total number of receptor complexes, as well as related to the percentages of labeled neurons (25 % for CN, 24 % for Put, 37 % for AcbCo and 56 % for AcbSh) and the mean single-cell densities (19.5 for CN, 20.5 for Put, 36.3 for AcbCo and 60.4 for AcbSh). In other words, the highest abundance of D1–D2 receptor heteromers was consistently found at the level of the AcbSh, in terms of number of receptors as well as in both the percentages of labeled neurons and in the mean single-cell densities (Fig. 5). Moreover, it is also worth noting that the MPTP-treated animal group (without dyskinesia) showed a moderate increase in the total number of D1–D2 receptor heteromers in the CN and Put nuclei. These increases correlated with higher values for single-cell densities of heteromers in the striatal areas showing more severe dopaminergic depletion compared to AcbCo and AcbSh, the latter striatal territories being mainly innervated by neurons located in the ventral tegmental area, a dopaminergic group less vulnerable to MPTP intoxication than the substantia nigra pars compacta (Fig. 1). Furthermore and regarding the obtained values for total number of D1–D2 receptor heteromers as well as for single-cell densities in the CN and Put from dyskinetic animals, these values did not differ numerically from control levels as a result of the chronic treatment with levodopa (Fig. 5).

### Two types of D1–D2 receptor heteromers

**Fig. 6:**
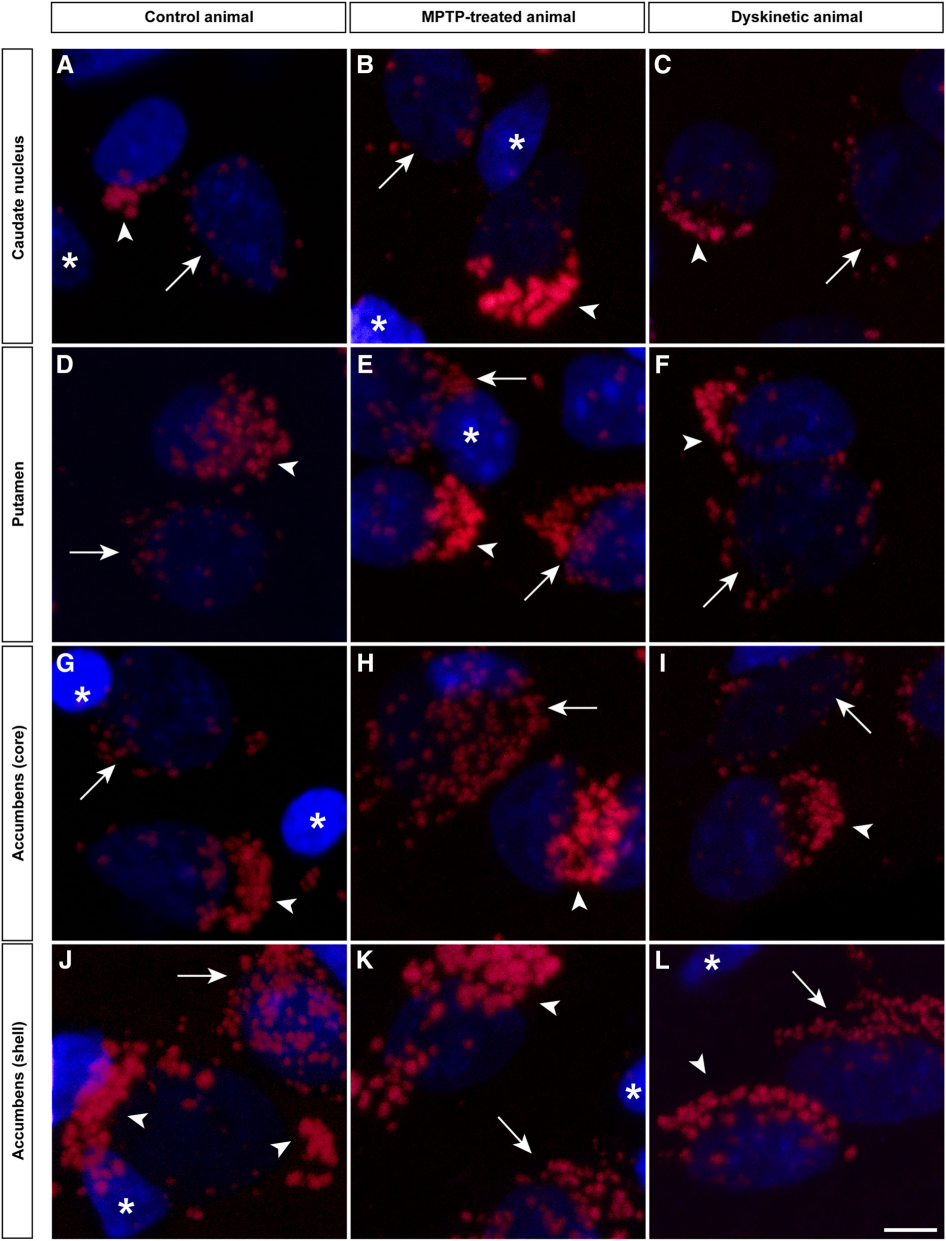
Two different types of D1–D2 receptor heteromers. The careful inspection of the material revealed the presence of D1–D2 receptors of different sizes, as observed within all striatal subdivisions and across all experimental groups. Unexpectedly, neighboring neurons often showed PLA signals of two different sizes, likely reflecting the presence of both individual D1–D2 receptor heteromers and macromolecular structures made of a number of individual receptor heteromers, grouped together.* Arrows* and* arrowheads* indicate neurons showing individual and macromolecular D1–D2 heteromers, respectively. Glial cells lacking D1–D2 heteromers are marked with asterisks. *Scale bar* 5 mm in all *panels*

Besides the obtained evidence on the presence of D1–D2 receptor heteromers, two different types of D1–D2 receptor heteromers were consistently found throughout all striatal territories and across all different animal groups. According to the foundations of the PLA method, each single protein-to-protein interaction will finally be visualized as a red spot, here accounting for a pair of D1 and D2 receptors in close proximity. However, after inspection of the labeled material, the existence of two types of PLA-labeled red spots was clearly noticed. Although individual D1–D2 receptor heteromers were by far the most commonly observed, neurons expressing a “macromolecular” type of D1–D2 receptor heteromers were constantly found intermingled with striatal neurons expressing individual D1–D2 receptor heteromers, without any apparent territorial preferential distribution within the striatum (Fig. 6), and were, indeed, observed in all animal groups. Furthermore and in keeping with what was observed for individual D1–D2 receptor heteromers, “macromolecular” heteromers were only found surrounding neuronal nuclei, without any labeling in glial cells.

**Fig. 7:**
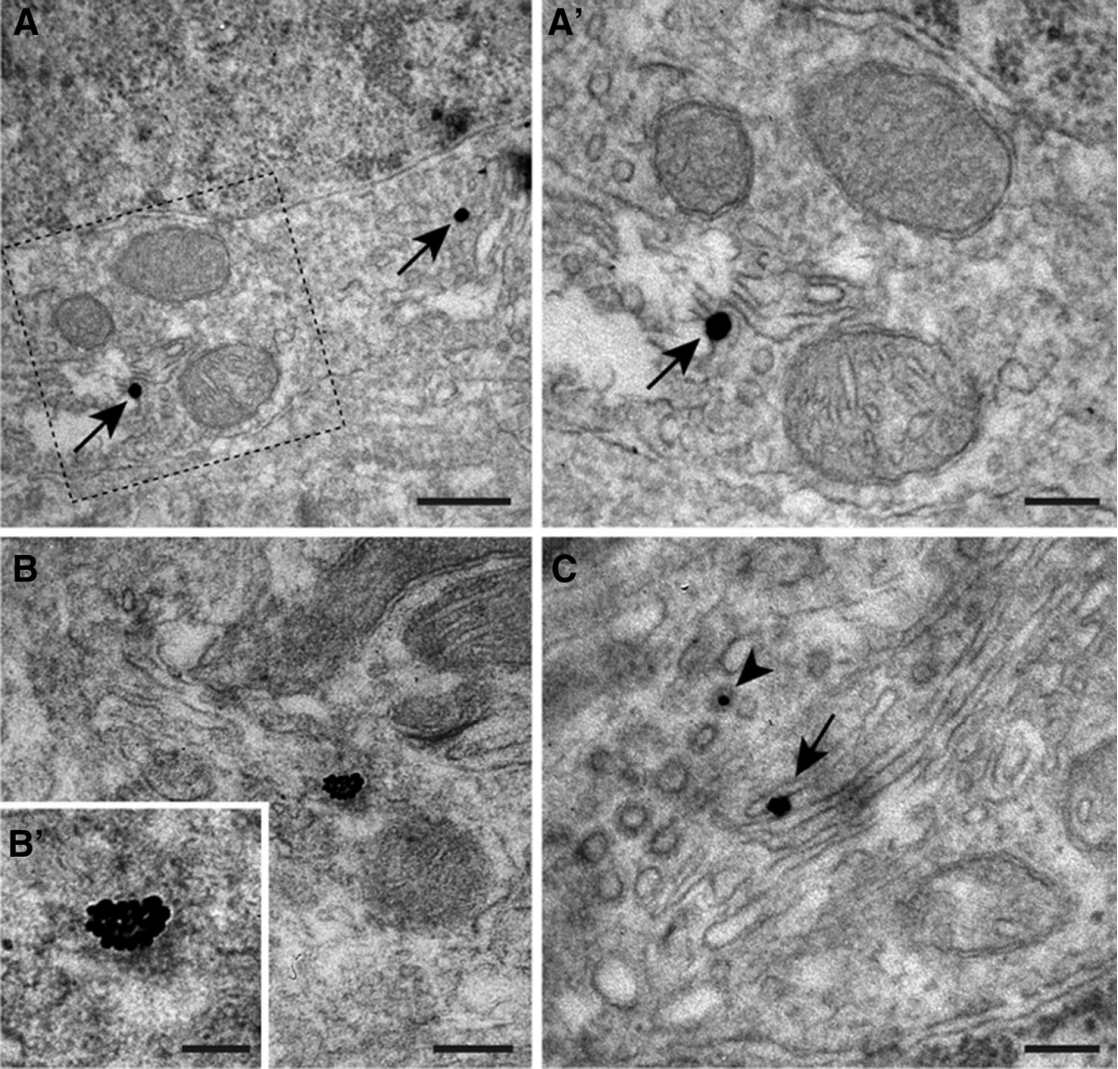
Ultrastructural detection of D1–D2 receptor heteromers. Taking the PLA method to the electron microscopy confirmed the presence of D1–D2 receptor heteromers in striatal neurons. This procedure also enabled the demonstration of two different types of D1–D2 receptor heteromers, comprising individual heteromers (**a** and **a**′, taken from the caudate nucleus) as well as macromolecular structures made by the addition of a substantial number of receptor heteromers (**b** and **b**′; accumbens shell). Furthermore, D1–D2 receptor heteromers were found in the cisternae of the Golgi apparatus (*arrow* in **c**) as well as within secretory vesicles of the *trans* face of the Golgi apparatus (*arrowhead* **c**). This finding represents the first demonstration showing that D1–D2 receptor heteromers are already synthesized and preassembled together in the Golgi apparatus, instead of being synthesized separately and later assembled at the level of the cellular membrane. *Scale bar* 500 nm in **a**, 200 nm in **a**′, 500 nm in **b**, 100 nm in **b**′ and 200 nm in **c**

These findings prompted us to investigate the idea that besides individual D1–D2 receptor heteromers, these receptor heteromers might also be sometimes arranged in macromolecular structures made of a number of D1–D2 receptor heteromers, grouped together. This idea was corroborated by taking the PLA assay to the ultrastructural level. As illustrated in Fig. 6, some striatal neurons showed PLA-labeled structures made of the addition of a substantial number of individual D1–D2 receptor heteromers (often comprising between 20 and 30 D1–D2 receptor heteromers). Moreover, ultrastructural evidence showed that D1–D2 receptor heteromers were, indeed, already assembled together during synthesis, since these receptor heteromers were found within the cisternae of the Golgi apparatus as well as in secretory vesicles in the *trans* face of the Golgi apparatus (Fig. 7).

### Expression of D1–D2 receptor heteromers in different types of striatal neurons

In an attempt to identify the identities of neurons expressing either individual or “macromolecular” D1–D2 receptor heteromers, the PLA technique was combined with either retrograde tract-tracing experiments disclosing striatofugal neurons or with a number of markers characterizing different types of striatal interneurons.

**Fig. 8:**
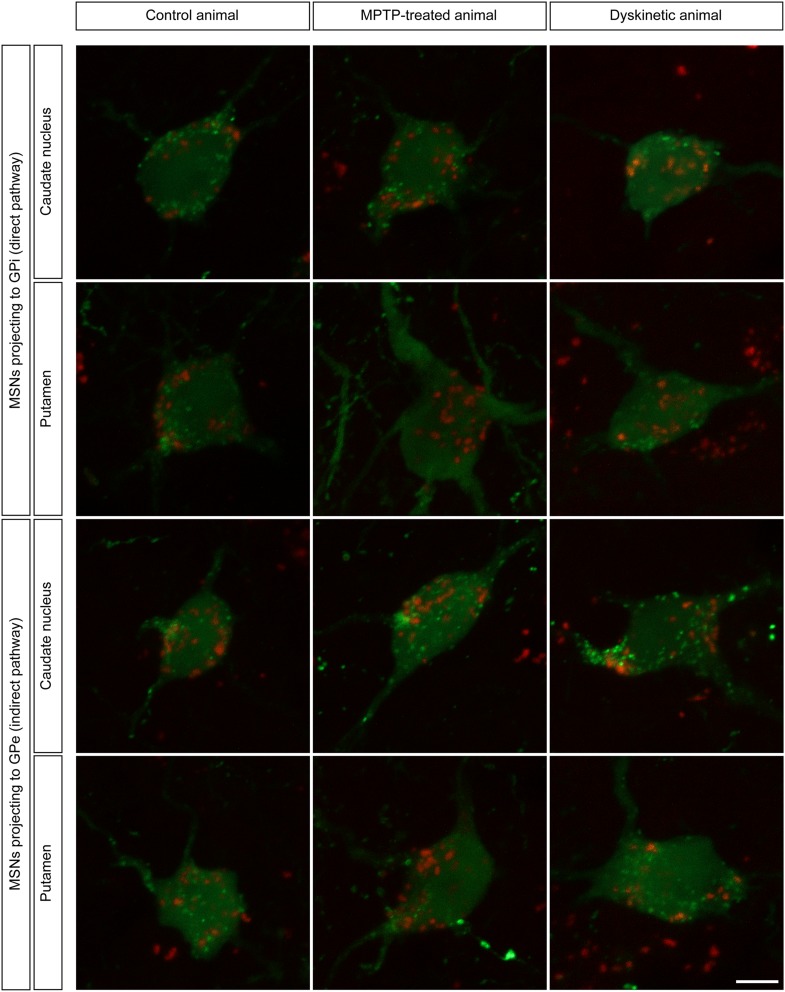
Expression of D1–D2 receptor heteromers in striatofugal neurons. Following the delivery of the retrograde tracer biotinylated dextran amine (BDA) into either the internal or external subdivisions of the globus pallidus (GPi and GPe, respectively), neurons projecting through the direct or the indirect pathway were identified within the caudate and putamen nuclei (*green* channel). The combination of BDA retrograde tracing together with the PLA protocol enabled the unequivocal demonstration of the expression of D1–D2 receptor heteromers (*red color*) within both subtypes of striatopallidal-projecting neurons. *Scale bar* 5 mm in all *panels*

Striatal projection neurons giving rise to either the direct or the indirect basal ganglia pathways were unequivocally identified following the delivery of the retrograde tracer BDA 10 kDa into the GPi and GPe nuclei, respectively, in six macaques (two control, two Parkinsonian and two dyskinetic). Tracer leakage through the needle tract was not observed in any of the BDA-injected macaques (Fig. 1). A moderate number of BDA-labeled neurons were observed throughout central and dorsolateral territories of the post-commissural putamen as well as in the body of the caudate nucleus. The combination of the PLA assay with the histofluorescent detection of transported BDA enabled the unequivocal demonstration that a subpopulation of BDA-labeled striatopallidal-projecting neurons (innervating either the GPi or the GPe nuclei) expressed the individual type of D1–D2 receptor heteromers, as observed in control, MPTP-treated and dyskinetic animals (Fig. 8). In keeping with the observed single-cell densities of D1–D2 receptor heteromers, striatopallidal-projecting neurons in Parkinsonian animals displayed a larger amount of heteromers when compared to those observed both in control and dyskinetic macaques.

**Fig. 9:**
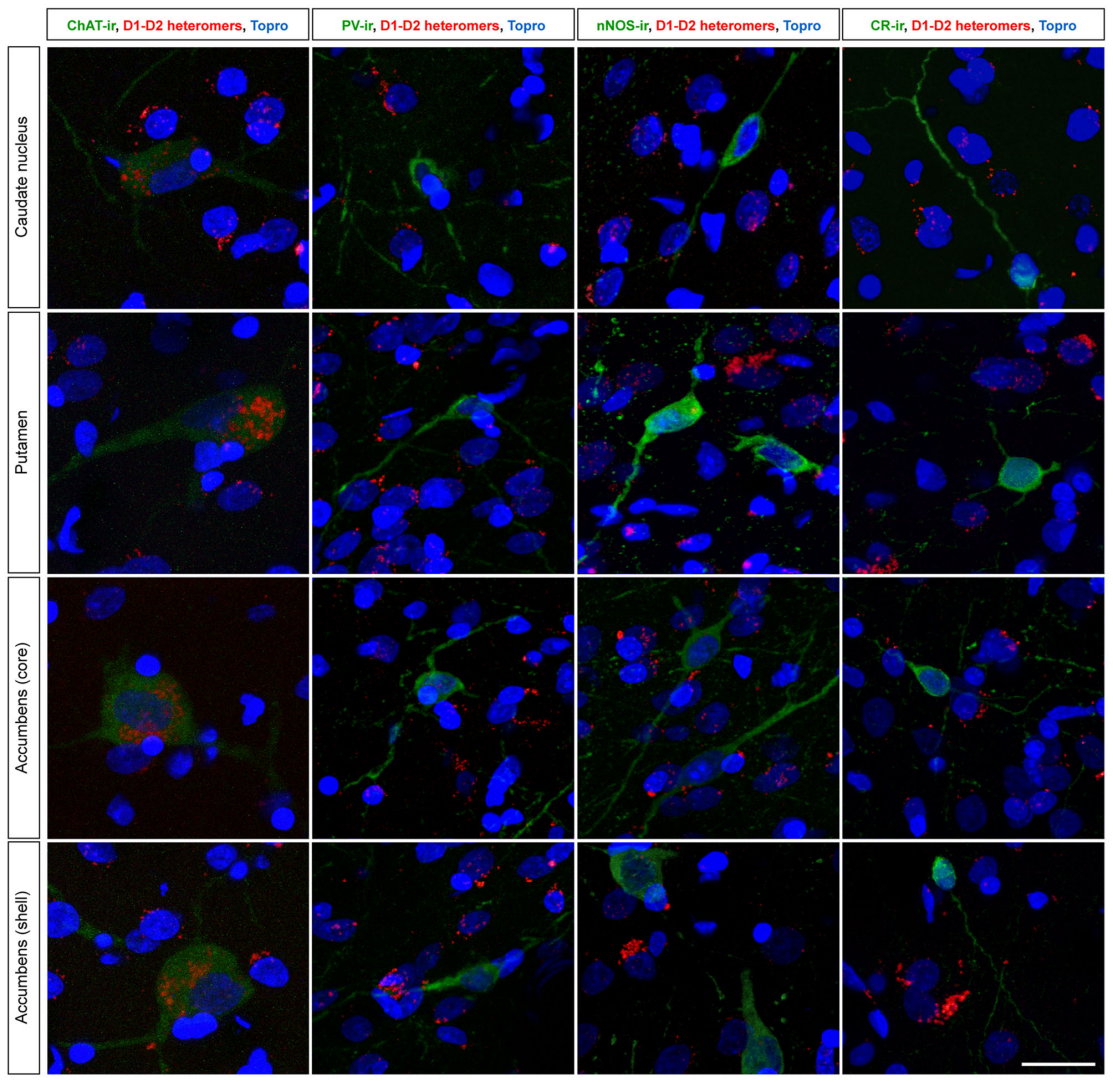
Expression of D1–D2 receptor heteromers in different types of striatal interneurons. Here, the immunofluorescent detection of the four main different types of striatal interneurons (e.g., those positive for ChAT, parvalbumin, nNOS and calretinin) was combined with the PLA technique and further counterstained with Topro-3. Obtained images clearly showed that cholinergic interneurons are the only ones expressing macromolecular D1–D2 receptor heteromers, whereas striatal interneurons other than cholinergic completely lack neither individual nor macromolecular D1–D2 receptor heteromers. *Scale bar* 20 mm in all *panels*

Bearing in mind that projection neurons lacked “macromolecular” D1–D2 receptor heteromers, the PLA assay was combined with a number of markers identifying the main different types of striatal neurons, namely cholinergic (ChAT+), parvalbumin (PV+), calretinin (CR+) and nitrergic (nNOS+) interneurons. After extensive search throughout all striatal divisions, ChAT + neurons were the only ones expressing the “macromolecular” version of D1–D2 receptor heteromers. All the remaining classes of striatal interneurons were never found to express neither the individual nor the “macromolecular” types of D1–D2 receptor heteromers (Fig. 9).

## Discussion

Evidence is provided here showing that D1–D2 receptor heteromers are expressed within all striatal territories examined (CN, Put, AcbCo and AcbSh), both in control macaques as well as in MPTP-treated macaques (with or without levodopa-induced dyskinesia). In keeping with existing knowledge, the highest abundance of D1–D2 receptor heteromers was found in the AcbSh, followed by the AcbCo, the Put nucleus and the CN. Moreover, two different types of heteromers were found, comprising individual D1–D2 receptors that were typically found in both striatal direct and indirect pathway projection neurons, and “macromolecular” D1–D2 receptor complexes that were only observed in cholinergic interneurons. Striatal interneurons other than cholinergic completely lacked any type of D1–D2 receptor heteromers. Furthermore, when compared to control animals, a higher number of D1–D2 receptor heteromers was found in the CN and Put nuclei from MPTP-treated animals (without levodopa treatment), reflecting increased single-cell density of heteromers. Finally, observed increases returned back to control levels in animals showing levodopa-induced dyskinesia. It is worth noting that the small sample size (two macaques per group) has precluded statistical detection of inter-group variations.

### Technical considerations

GPCR heteromeric complexes have been often studied in vitro and ex vivo by relying on a number of techniques (co-immunoprecipitation, BRET, FRET, competition binding assays) that indirectly demonstrate the presence of molecular interactions between each receptor forming the heteromeric complex. By taking advantage of these procedures, several GPCR heteromeric complexes have been characterized throughout the last decade, all of them exhibiting a number of biochemical and molecular properties different than those of each component receptor, when considered individually. Although these techniques—particularly when combined together—have proven to be instrumental in pushing ahead the characterization of the molecular properties for different types of GPCR heteromers, the availability of a procedure enabling the direct visualization of a given GPCR heteromer has long been considered as an unmet need. This pending gap has been properly fulfilled by the introduction of the so-called in situ proximity ligation assay (PLA; Söderberg et al. 2008). The PLA technique is based on the use of two primary antibodies (against each target receptor) followed by the use of two secondary antibodies covalently coupled to a pair of affinity oligonucleotide probes (a plus and a minus probe). Only when the target proteins are in a very close proximity (e.g., less than 17 nm), the probes do ligate and form templates for rolling circle amplification (Söderberg et al. 2008; Trifilieff et al. 2011). Hybridization of complementary fluorescently labeled oligonucleotides with the amplified DNA is then most commonly seen as a red dot of sub-micrometer size with fluorescent microscopy. In summary, the result from a PLA experiment is typically a number of discrete red fluorescent signals in various locations of the studied cells or tissue samples, each red dot thus representing a single protein–protein complex. Although the PLA technique cannot be used to prove the presence of molecular interactions between each component receptor forming a heteromeric complex (i.e., it only accounts for close proximity), the main added value is represented by the fact that the accurate localization of a given GPCR heteromer can be achieved in an unprecedented way.

Before starting to plan a PLA experiment, a number of demands should be taken into consideration, these including (a) availability of specific primary antibodies against each protein to be detected, (b) the two primary antibodies should have been raised in different animal species and (c) accurate selection of the most appropriate PLA-labeled secondary antibodies (plus and minus PLA probes). The most conventional PLA protocol relies on the so-called “secondary detection” that basically implies the choice of the Duolink in situ PLA detection kit, made of secondary antibodies tagged to complementary DNA plus and minus probes. Probes against mouse, goat and rabbit antibodies are the only ones currently available in the Olink/Sigma catalogue. Accordingly, one may encounter two different types of problems just in case that (a) antibodies against the two proteins to be detected by proximity are raised in the same animal species or (b) one of the primary antibodies is raised in an animal species other than mouse, goat or rabbit. To circumvent these limitations, Olink/Sigma provides potential users with the so-called Duolink in situ Probemaker kits (plus and minus) that basically enable the direct conjugation of the primary antibodies with the plus and minus PLA probes.

The well-established presence of D1–D2 receptor heteromers in rat striatum has recently been challenged (Frederick et al. 2015). Besides a number of molecular and biochemical assays, these authors took advantage of the PLA technique in an attempt to provide a final evidence of the lack of D1–D2 receptor heteromers. However, they have followed a somewhat unconventional PLA procedure, as follows: the detection of D2 receptors was accomplished using a rabbit anti-D2 primary antibody followed by an anti-rabbit PLA probe from the Duolink in situ PLA detection kit, whereas for the detection of D1 receptors, a rat anti-D1 primary antibody was chosen followed by an anti-rat PLA probe generated with the Duolink in situ Probemaker kit and a goat anti-rat IgG. Such a succinct description impedes the proper understanding of the conducted protocol, since a number of items required to be better explained. Briefly, it seems that a “secondary detection” was used for D2 receptors together with a “primary detection” (i.e., conjugating the rat anti-D1 antibody with the Probemaker kit) for the D1 receptor. Alternatively, it might be the case that the goat anti-rat IgG was the antibody being conjugated with the Probemaker kit. In this regard, the use of an anti-goat probe from the Duolink in situ PLA detection kit sounds as a much more appropriate choice. Otherwise, it remains unclear as to what was the ultimate reason sustaining the use of a goat anti-rat IgG within the protocol. By performing the PLA protocol this way, the authors reported the presence of strong nuclear labeling that was claimed to be nonspecific even when considering that the nuclear signal was, by far, more prominent than the PLA signal in itself as obtained in mice overexpressing D1 and D2 receptors (Frederick et al. 2015). Furthermore, the reported non-nuclear PLA signal even in the mice overexpressing D1 and D2 receptors did not localize around the neuronal nuclei—as observed here—but were diffusely distributed. In all the PLA experiments conducted here in non-human primates, as well as in former studies dealing with different types of GPCR heteromers (Navarro et al. 2008; Bonaventura et al. 2014; Martinez-Pinilla et al. 2014, 2015; Farré et al. 2015; Sierra et al. 2015), the presence of PLA signals in the cell nuclei has never been observed. In summary, we strongly believe that the selected PLA protocol has prevented Frederick et al. (2015) to accurately detect D1–D2 heteromers.

Here, we have chosen what we think is the most appropriate choice for the PLA protocol when there is no anti-rat PLA probe available within the Duolink in situ PLA detection kit. Briefly, the Duolink Probemaker kit was used to prepare an anti-D2 PLA probe (plus probe) as well as to prepare an anti-D1 probe (minus probe), e.g., each primary antibody was directly conjugated with a PLA probe according to the guidelines provided by the manufacturer.

### Striatal expression of D1–D2 receptor heteromers

D1 and D2 receptors are considered to be segregated in striatofugal neurons giving rise to the direct and indirect basal ganglia pathways, respectively (Gerfen et al. 1990; Le Moine and Bloch 1995; Aubert et al. 2000; Bertran-Gonzalez et al. 2010), with minimal percentages of striatal neurons expressing both receptors. However, such a potential overlap of D1 and D2 receptors within striatal medium-sized spiny neurons (MSNs) is still a matter of debate. Depending on the specificity of the antibodies used to characterize D1- and D2-containing MSNs as well as on the different methods being conducted, substantial differences for D1–D2 co-labeling were reported, ranging from an almost complete co-expression of both receptors in striatal neurons (Aizman et al. 2000), down to moderate (15–20 %; Deng et al. 2006) or to low percentages such as 7 % (Perrreault et al. 2010), or to even lower in dorsal striatum (Gangarossa et al. 2013a, b). In ventral striatum, higher levels of co-expression have been shown, up to 38 % (Gangarossa et al. 2013b). Moreover, although it is often assumed that D1- and D2-expressing MSNs are physically intermingled in a mosaic-like distribution, a number of regional differences throughout the rostrocaudal axis of the striatum have been reported (Gangarossa et al. 2013a).

Our current data showed that the highest expression of D1–D2 receptor heteromers in terms of total number of PLA signals, mean single-cell density and percentage of neurons exhibiting D1–D2 labeling was found in the AcbSh, a striatal area known to be the one with the highest co-expression of D1 and D2 receptors in rat and mouse (Hasbi et al. 2009; Perrreault et al. 2010, 2011, 2012; Gangarossa et al. 2013b). Moreover, the highest abundance of D1–D2 receptor heteromers in the AcbSh was maintained throughout all experimental groups examined, without any apparent influence of neither nigrostriatal dopaminergic denervation nor chronic dopamine replacement. It is also worth noting that the high co-expression levels of D1 and D2 receptors in the AcbSh has paved the way for suggesting the existence of a third basal ganglia output pathway, different from the traditionally considered direct and indirect pathways (Perreault et al. 2011). Moreover, the CN was found to be the striatal territory with the weakest expression of D1–D2 receptor heteromers, together with the lowest percentage of labeled neurons as well as the mean single-cell density, the latter parameter being very low (19.8 D1–D2 heteromers per labeled cell). Roughly similar mean values were obtained in dyskinetic animals (19.5), whereas the MPTP treatment (without levodopa administration) induced a moderate increase in the mean single-cell values (27.6). We believe that these data fit well with those available from the literature reporting that the highest D1 and D2 receptor co-expression takes place in the accumbal subregions.

Indeed, it is also important to stress the fact that the PLA assay merely detects two proteins (here D1 and D2 receptors) located in very close physical proximity, and therefore, this technique does not provide any clues on the total number of D1 and D2 receptors, when considered separately. In this regard, striatal neurons lacking PLA labeling would be likely to express only either D1 or D2 receptors. Accordingly, the obtained values of unlabeled neurons (approximately 74 % for CN and Put, 64 % for AcbCo, and 44 % for AcbSh), together with the lowest D1–D2 single-cell densities for CN-and Put-labeled neurons fit well within a very low co-expression of D1 and D2 receptors in dorsal striatal areas. Furthermore, it is also worth noting that the moderate increases observed in the total number of D1–D2 receptor heteromers in MPTP-treated primates did not reflect a change in the percentage of labeled vs. unlabeled neurons but a moderate increase in single-cell densities. In other words, it seems to be the case that dopaminergic depletion induced the synthesis of more D1–D2 receptor heteromers (also very likely when considering individual D1 and D2 receptors) within the striatal neurons already containing these heteromeric complexes, instead of being synthesized de novo within striatal neurons not formerly expressing D1–D2 receptor heteromers.

Finally, it is worth noting that a number of changes in D1 and D2 receptor expression have been found in different clinical conditions. Most of the available evidence suggest the presence of dopamine receptor supersensitivity (Ding et al. 2015), particularly increased D1 receptor-mediated transmission as a mechanism underlying levodopa-induced dyskinesia (Aubert et al. 2005). Besides these receptor supersensitivities, it seems that levodopa-induced dyskinesia results in increased availability of D1 receptors at the plasma membrane, with modest changes considering the cytoplasmic *vs*. membrane location when considering D2 receptors (Guigoni et al. 2007). Data presented here showed that D1–D2 receptor heteromer expression in dyskinetic conditions is roughly similar to control conditions, both in terms of number of heteromers as well as when considering single-cell densities. However, bearing in mind that the PLA technique only enables the detection of D1–D2 heteromers, we cannot provide further clues on changes within D1 and D2 receptors, when considered separately.

Taken together, these data are in agreement with earlier knowledge on D1–D2 receptor heteromerization in rat striatum but provide the first demonstration in non-human primate striatum with characterization of distinct neuronal phenotypes expressing D1–D2 heteromers, thus supporting the further functional analysis and development of selective ligands targeting dopamine receptor heteromeric complexes with reduced off-target effects in basal ganglia-related diseases. The consideration of D1–D2 receptor heteromers as putative novel pharmacological targets will pave the way for novel therapeutic developments on a number of dopamine-related brain diseases.
